# The risk of alcohol use disorders in offspring who had hyperactivity problems: The ALSPAC study

**DOI:** 10.1192/j.eurpsy.2023.361

**Published:** 2023-07-19

**Authors:** B. A. Dachew, R. Alati

**Affiliations:** School of Population Health, Curtin University, Perth, Australia

## Abstract

**Introduction:**

There is a paucity of population-based longitudinal studies examining the associations between childhood behavioural problems and alcohol use disorders in later life.

**Objectives:**

This study aimed to examine the association between hyperactivity/inattention problems in early adolescence and the risk of alcohol use disorders in young adulthood.

**Methods:**

We used data from the Avon Longitudinal Study of Parents and Children (ALSPAC), a population-based prospective cohort based in Bristol, United Kingdom. Hyperactivity/inattention problems at 11 years of age were measured using the Strengths and Difficulties Questionnaire (SDQ). Logistic regression analyses were used to examine associations. E-values (E) were calculated to estimate the extent of unmeasured confounding.

**Results:**

Hyperactivity/ inattention problems in early adolesce were associated with a 1.75-fold increased risk of any alcohol use disorders (OR = 1.75, 95% CI: 1.20-2.56; E= 2.90, CI: 1.69) and a 4-fold increased risk of severe alcohol use disorders at age 24 (OR = 4.35, 95% CI: 2.00 – 9.47; E= 8.17, CI: 3.58). We also found a 2.09 (OR = 2.09, 95 % CI: 1.24-3.53; E= 3.60, CI: 1.79) and 1.63-fold (OR = 1.63, 95% CI: 1.07 – 2.49; E= 2.64, CI: 1.34) increased risk of alcohol dependence symptoms and alcohol abuse symptoms at age 24 in offspring who had hyperactivity problems at age 11, respectively.

**Conclusions:**

Hyperactivity/ inattention problems in early adolesce were associated with an increased risk of alcohol use disorder symptoms in adulthood, even when controlling for conduct problems. Associations did not appear to differ by gender and unmeasured or unknown confounders were unlikely to alter the observed associations.

**Disclosure of Interest:**

None Declared

